# Urinary benzyl alcohol and hippuric acid in workers exposed to benzyl alcohol during paint-stripping work

**DOI:** 10.1093/joccuh/uiae059

**Published:** 2024-10-04

**Authors:** Kenta Ishii, Akito Takeuchi, Masami Shimada, Hiromi Momokawa, Tomiko Tashiro, Ai Yamada, Kumiko Arai, Akira Namera, Kenji Yamamuro, Koichi Kato, Toshihiro Kawamoto, Ginji Endo

**Affiliations:** Osaka Occupational Health Service Center, Japan Industrial Safety and Health Association, 2-3-8 Tosabori, Nishi-Ku, Osaka 550-0001, Japan; Technical Support Department, Japan Industrial Safety and Health Association, 5-35-2 Shiba, Minato-Ku, Tokyo 108-0014, Japan; Occupational Health Research and Development Center, Japan Industrial Safety and Health Association, 5-35-2 Shiba, Minato-Ku, Tokyo 108-0014, Japan; Laboratory of Environmental Toxicology and Carcinogenesis, School of Pharmacy, Nihon University, 7-7-1 Narashinodai, Funabashi, Chiba 274-8555, Japan; Osaka Occupational Health Service Center, Japan Industrial Safety and Health Association, 2-3-8 Tosabori, Nishi-Ku, Osaka 550-0001, Japan; Occupational Health Research and Development Center, Japan Industrial Safety and Health Association, 5-35-2 Shiba, Minato-Ku, Tokyo 108-0014, Japan; Occupational Health Research and Development Center, Japan Industrial Safety and Health Association, 5-35-2 Shiba, Minato-Ku, Tokyo 108-0014, Japan; Osaka Occupational Health Service Center, Japan Industrial Safety and Health Association, 2-3-8 Tosabori, Nishi-Ku, Osaka 550-0001, Japan; Osaka Occupational Health Service Center, Japan Industrial Safety and Health Association, 2-3-8 Tosabori, Nishi-Ku, Osaka 550-0001, Japan; Department of Pathophysiological Laboratory Sciences, Field of Radiological and Medical Laboratory Sciences, Nagoya University Graduate School of Medicine, 1-1-20 Daiko-minami, Higashi-Ku, Nagoya 461-8673, Japan; Occupational Health Research and Development Center, Japan Industrial Safety and Health Association, 5-35-2 Shiba, Minato-Ku, Tokyo 108-0014, Japan; Department of Forensic Medicine, Graduate School of Biomedical and Health Sciences, Hiroshima University, 1-2-3 Kasumi, Minami-Ku, Hiroshima 734-8553, Japan; Occupational Health Research and Development Center, Japan Industrial Safety and Health Association, 5-35-2 Shiba, Minato-Ku, Tokyo 108-0014, Japan; Laboratory of Environmental Toxicology and Carcinogenesis, School of Pharmacy, Nihon University, 7-7-1 Narashinodai, Funabashi, Chiba 274-8555, Japan; Occupational Health Research and Development Center, Japan Industrial Safety and Health Association, 5-35-2 Shiba, Minato-Ku, Tokyo 108-0014, Japan; Osaka Occupational Health Service Center, Japan Industrial Safety and Health Association, 2-3-8 Tosabori, Nishi-Ku, Osaka 550-0001, Japan

**Keywords:** benzyl alcohol, biological monitoring, gas chromatography–mass spectrometry, occupational exposure, urine, worker

## Abstract

Objective: We aimed to develop a reliable gas chromatography–mass spectrometry (GC–MS) method for detecting urinary benzyl alcohol (BeOH) concentrations and assess the suitability of urinary BeOH as a biomarker for occupational BeOH exposure.

Methods: Thirteen male participants exposed to BeOH during paint-stripping work provided preshift and postshift urine samples, and their personal exposure concentrations were measured. Meanwhile, a control group of 10 nonexposed workers contributed urine samples. The newly developed GC–MS method met regulatory guidelines.

Results: The personal exposure concentrations of BeOH ranged from 8.4 to 45.2 mg/m^3^. Postshift urine samples from exposed participants showed significant BeOH and hippuric acid (HA) concentration increases compared with preshift samples (BeOH, post-/pre-shift geometric mean [GM] ratio = 7.5-7.8, *P* < .001; HA, post-/pre-shift GM ratio = 4.3-4.5, *P* < .001). These levels were considerably higher than those in postshift samples from the nonexposed control group (BeOH, exposed-/nonexposed-workers GM ratio = 14.8-19.0, *P* < .001; HA, exposed-/nonexposed-workers GM ratio = 12.1-15.3, *P* < .001), even after urine density correction.

Conclusions: Urinary BeOH and HA can serve as potential biomarkers of occupational exposure to BeOH. More specifically, BeOH might serve as a biomarker superior to HA because it is apparently less influenced by confounding factors such as dietary intake and genetic polymorphism of low-*K*_m_ aldehyde dehydrogenase (ALDH2). The findings will improve workplace safety measures and protocols, assisting health care professionals in diagnosing and managing exposure-related health issues, thereby potentially reducing the risk of occupational exposure to BeOH.

## Teaser

Key points

- **What is already known on this topic:** Recently in Japan, the frequency of occupational poisoning accidents among workers exposed to BeOH-containing paint removers has increased. To the best of our knowledge, no studies have focused on the biological monitoring of occupational exposure to BeOH.
- **What this study adds:** Urinary BeOH and HA can serve as potential biomarkers of occupational exposure to BeOH.
- **How this study might affect research, practice, or policy:** The findings will improve workplace safety measures and protocols, assisting health care professionals in diagnosing and managing exposure-related health issues, thereby potentially reducing the risk of occupational exposure to BeOH.

## 1. Introduction

Benzyl alcohol (BeOH, CAS No. 100-51-6) is a colorless liquid characterized by a boiling point of 204.7°C and a saturated vapor pressure of 3 Pa (20°C).^1^ BeOH is applied in paint removers and cosmetics, acts as a curing agent in epoxy resins, serves as a solvent in waterborne coatings, and functions as a preservative and flavoring in food processing.^1^^,^^2^ For BeOH, the Japan Society for Occupational Health (JSOH)^1^ and the Deutsche Forschungsgemeinschaft (DFG)^2^ have proposed an occupational exposure limit-ceiling (OEL-C) of 25 mg/m^3^ and a maximum workplace concentration (Maximale Arbeitsplatz-Konzentration, MAK) of 22 mg/m^3^, respectively. However, the American Conference of Governmental Industrial Hygienists has not proposed any OEL for BeOH. Notably, the DFG^2^ has classified BeOH as a substance that can be absorbed through the skin in toxicologically significant amounts.

Recently in Japan, the frequency of occupational poisoning accidents among workers exposed to BeOH-containing paint removers has increased.^3^^-^^5^ To prevent such accidents, in 2020, the Ministry of Health, Labor and Welfare (MHLW)^5^ of Japan amended the Order for Enforcement of Industrial Safety and Health Act and the Ordinance on Industrial Safety and Health, ensuring effective management of BeOH by labeling of hazards, providing a safety data sheet, and performing risk assessment. Subsequently, in 2021, the MHLW commissioned the Japan Industrial Safety and Health Association (JISHA) to conduct a field survey and assess the BeOH exposure levels of workers engaged in the process of removing bridge paint film. This survey included personal exposure monitoring and biological monitoring.^6^

**Figure 1:**
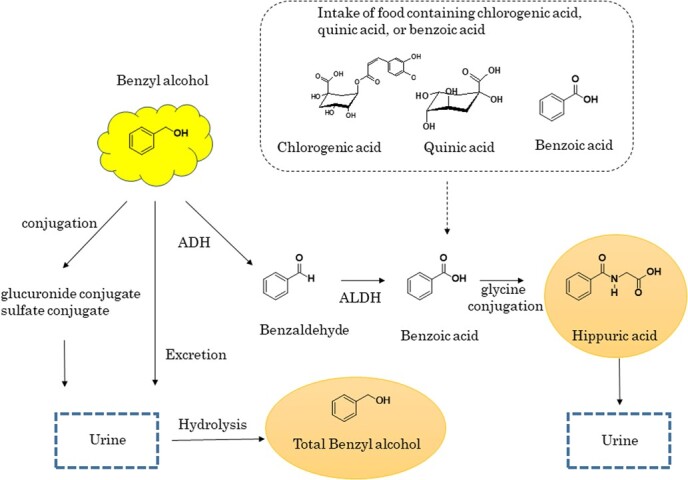
Metabolism of benzyl alcohol in humans.^7^ ADH, alcohol dehydrogenase; ALDH, aldehyde dehydrogenase.

Biological monitoring is useful for assessing the level of internal exposure of a worker to a harmful substance because it reflects the total amount absorbed into the body, regardless of the exposure route. Nonetheless, to the best of our knowledge, no studies have focused on the biological monitoring of occupational exposure to BeOH. Most BeOH absorbed into the human body is rapidly oxidized via benzaldehyde to benzoic acid, subsequently conjugating with glycine to form hippuric acid (HA), and ultimately excreted through the urine (Figure 1).^7^ Although urinary HA can serve as a suitable biomarker for BeOH exposure, the background concentration of urinary HA, primarily attributable to diet, may obscure the contribution of occupational exposure to BeOH. Therefore, we directed our attention toward urinary BeOH as a relevant biomarker because it is less affected by diet.

This study aimed to develop a reliable and robust gas chromatography–mass spectrometry (GC–MS) method for determining urinary BeOH by improving the method reported by Kawai et al^8^ and to evaluate whether urinary BeOH and HA are indicative biomarkers of occupational exposure to BeOH. To this end, we used the newly developed method for analyzing the BeOH concentration in urine samples collected from BeOH-exposed workers during the aforementioned field survey conducted by JISHA. The results were compared with their corresponding HA outcomes. Furthermore, the relationship between the personal exposure concentrations of BeOH and the concentrations of BeOH or HA in urine samples was examined.

## 2. Methods

### 2.1. Subjects and urine collection

The study involved 13 Japanese workers—all males—engaged in the process of removing paint films from bridges using paint removers containing BeOH. The paint remover contains BeOH (35%-65%) but not toluene. For comparison, 1 female and 9 male office workers from a different company with no BeOH exposure participated as the control group. All workers employed protective clothing, masks, and gloves. No personal data such as age, smoking, and alcohol consumption habits, were available. Urine samples were collected preshift and postshift from each participant on the same day. Thereafter, the samples were stored at −20°C in darkness until further analysis. The JISHA’s Ethics Committee approved the study (approval number 202103). All participants provided their written informed consent prior to sample collection. The blank urine used for the calibration curve was obtained from the control group.

### 2.2. Personal exposure monitoring and analysis of BeOH

Personal exposure monitoring was conducted on the same day as urine was collected. Sampling and analysis were carried out using the method developed by Aono et al.^9^ To determine the time-weighted average level of workers’ exposure to BeOH, samplers were placed near each worker’s breathing zone during the entire work shift (8 hours).

### 2.3. Analysis of urinary BeOH

#### 2.3.1. Materials

BeOH and methyl tert-butyl ether (MTBE) were purchased from Tokyo Chemical Industry (Tokyo, Japan). Diisopropyl ether (DIPE) and 50% sodium hydroxide solution were purchased from FUJIFILM Wako Pure Chemical Corporation (Osaka, Japan). Benzyl-d_7_ alcohol (BeOH-d_7_) was procured from C/D/N Isotopes Inc (Quebec, Canada) and used as an internal standard (I.S.). Acetonitrile, potassium dihydrogen phosphate, and hydrochloric acid (HCl) were sourced from Kanto Chemical Co, Inc (Tokyo, Japan). All reagents were superior to analytical grade and used without any further purification. A 100 mM phosphate buffer solution was prepared by dissolving potassium dihydrogen phosphate in ultrapure water and adjusting the pH to 7.0 with 50% sodium hydroxide solution. Working standard solutions of BeOH (5, 10, 25, 50, 100, 250, 500, and 1000 μg/L), each containing the I.S. (500 μg/L), were prepared in acetonitrile and stored at 4°C in darkness.

#### 2.3.2. Instruments

A gas chromatograph (GC) system (6890GC; Agilent Technologies, Palo Alto, CA, USA) equipped with a DB-Heavy WAX capillary column (20 m × inner diameter [I.D.] 0.18 mm; film thickness: 0.18 μm) (Agilent Technologies, Palo Alto, CA, USA) and a mass spectrometer detector (MS; 5793 N; Agilent Technologies, Palo Alto, CA, USA) fitted with a quadrupole were employed to perform the analysis. Helium (purity: >99.999%; Iwatani fine gas, Osaka, Japan) was employed as a carrier gas at a flow rate of 1.0 mL/min. The inlet temperature was set at 250°C, and the pulsed split mode was adopted (injection volume: 1 μL; split ratio: 10:1; pulse pressure: 19.5 psi; pulse time: 1 minute). The oven temperature program was adjusted as follows: initial temperature of 40°C, hold for 1 minute and then ramp at 15°C/min to 280°C. The transfer line temperature was maintained at 280°C. The operating conditions of the MS included the electron ionization mode at an electron energy of 70 eV, an ion source temperature of 230°C, and a quadrupole temperature of 150°C. Furthermore, qualitative analysis was conducted in scan mode with a scanned mass range of mass-to-charge ratio (*m*/*z*) 35-500. Quantitative analysis was conducted in the selected ion monitoring mode, with the quantifier ion and qualifier ion at *m*/*z* 108 and 79 for BeOH and *m*/*z* 85 and 115 for the I.S., respectively.

#### 2.3.3. Sample preparation

The HCl hydrolysis procedure was conducted according to the Kawai method.^8^ Urine (5 mL), I.S. (500 μg/L, 50 μL), and HCl (35.0% - 37.0%, 0.5 mL) were added to a screw-capped glass test tube, which was vortexed for 1 minute and then heated in a boiling water bath for another 30 minutes. After cooling to room temperature (range of 23.0°± 2.0°C), MTBE (2 mL) and NaCl (2 g) were added to the test tube. The sample was vortexed for 1 minute and centrifuged at 1870 *g* for 10 minutes. An aliquot (1 mL) of the upper layer (extraction solution layer) was transferred to another glass test tube and added with the phosphate buffer solution (pH 7, 100 mM, 1 mL). After vortexing (1 minute) and centrifugation (1870 *g*, 10 minutes), the upper layer (1 μL) was injected into the GC–MS. If the BeOH concentration in the urine samples exceeded the calibration range, the samples were appropriately diluted and reanalyzed. Samples with high concentrations were analyzed by dilution with water and blank urine, and comparable results were obtained, suggesting no matrix influence.

#### 2.3.4. Method validation

The method validation was performed in accordance with the MHLW guideline and the US Food and Drug Administration guidance.^10^^,^^11^ To plot the calibration curve, standard urine samples at 8 concentrations (5, 10, 25, 50, 100, 250, 500, and 1000 μg/L) were generated by spiking working standard solutions into blank urine. These samples, prepared in triplicate, were analyzed using the procedure described above. Calibration curves were drawn by plotting the peak area ratios of BeOH (*m*/*z* 108) to the I.S. (*m*/*z* 85) with respect to the BeOH concentrations in standard urine samples. The reproducibility (defined as precision) and accuracy of the developed method were evaluated by analyzing the urine samples containing 4 varied BeOH concentrations (5, 25, 100, and 1000 μg/L) on the same day (5 replicates; intraday assay) and over 3 consecutive days (5 replicates each day; interday assay). Precision and accuracy were expressed as the relative SD and as the deviation from the nominal value, respectively. Recovery was calculated by comparing the peak area ratio of BeOH to the I.S. in urine samples spiked with standard solution before extraction versus samples spiked with standard solution into the urine extracts after extraction. The limit of detection (LOD) and limit of quantification (LOQ) were determined as the urinary BeOH concentrations equivalent to 3 and 10 times the baseline noise, respectively.

### 2.4. Analysis of urinary HA

Urinary HA was analyzed according to a previously reported method.^12^^,^^13^ The high-performance liquid chromatography (HPLC) system used was a Chromaster (Hitachi, Tokyo, Japan) equipped with a 5430-diode array detector. An Inertsil ODS-4 HP (100 × 3.0 mm; I.D.: 3 μm; GL Sciences Inc, Tokyo, Japan) separation column was used for the HPLC, with the flow rate set to 0.8 mL/min and the column temperature to 50°C. The mobile phase was a solution of [20 mM potassium dihydrogen phosphate containing 1.5 mM sodium 1-decanesulfonate]/acetonitrile (90/10; v/v). The detection wavelength was 225 nm. Samples that exceeded the calibration curve concentration were diluted and analyzed.

### 2.5. Statistical analyses

Assuming that personal exposure concentration of BeOH and urinary BeOH and HA concentrations adhere to a log-normal distribution, these data were log-transformed and statistically analyzed. Descriptive statistics for urinary BeOH and HA concentrations, such as geometric mean (GM) and geometric SDs, were evaluated separately for the pre- and post-shift urine samples collected from exposed and nonexposed workers. For urinary BeOH and HA, the data below the LOQ were set to half their respective LOQ (ie, BeOH: 2.5 μg/L; HA: 0.01 g/L). Urinary concentrations were recorded per observation (ie, without any correction for urine density) or after correction for creatinine (Cr) concentration or a specific gravity (SG) of urine of 1.020. In advance, we confirmed that the data for the 2 groups were normally distributed using the Shapiro-Wilk test. The *t* test was employed for paired samples to assess the differences between the urinary levels of each biomarker in the pre- and post-shift urine samples (ie, postshift vs preshift). Based on the Welch *t* test, the urinary levels of biomarkers in the pre- and post-shift urine samples of exposed workers were compared with those of nonexposed workers (ie, exposed workers vs nonexposed workers). Also, the F test was used to compare variances between groups. The relationship between the personal exposure concentrations of BeOH and BeOH or HA concentrations in urine samples was analyzed using simple regression. All reported *P* values were 2-sided and considered statistically significant at *P* < .05. All statistical analyses were conducted on a modified version of R commander—EZR Ver. 1.55 (Saitama Medical Center, Jichi Medical University, Saitama, Japan), which is a graphical user interface for R (R Foundation for Statistical Computing, Vienna, Austria)^14^—designed to incorporate frequently used statistical functions in biostatistics.

**Table 1:** Intra- and interday coefficients of variation of the proposed method.

**Spiked urine concentration, μg/L**	**Recovery (*n* = 5)**		**Intraday (*n* = 5)** ^**a**^			**Interday (*n* = 15)** ^**b**^		
	**Mean (SD), %**	**RSD, %**	**Mean (SD), μg/L**	**RSD, %**	**Accuracy, %**	**Mean (SD), μg/L**	**RSD, %**	**Accuracy, %**
5	99 (9.7)	9.7	4.61 (0.45)	9.7	99.2	4.67 (0.44)	9.4	93.3
25	106 (3.9)	3.7	23.75 (0.88)	3.7	95.0	23.81 (0.71)	3.0	95.2
100	104 (1.2)	1.2	97.05 (1.12)	1.2	100.2	98.97 (1.66)	1.7	99.0
1000	102 (0.4)	0.4	999.36 (3.79)	0.4	100.1	998.91 (5.81)	0.6	99.9

Abbreviation: RSD, relative standard deviation.

a Intraday reproducibility analysis was performed on a single day.

b Interday reproducibility analysis was performed over 3 consecutive days in 5 replicates.

## 3. Results

### 3.1. Analytical method improvement

To date, several methods have been reported for determination of BeOH in various matrices.^8^^,^^9^^,^^15^^-^^21^ However, to the best of our knowledge, the analytical method for urinary BeOH has been reported only by Kawai et al.^8^ Although we investigated the tried method as a preliminary analysis, it required further improvement. Therefore, we significantly re-examined their procedure in order to develop a more reliable methodology. Our enhancements include the changes of GC conditions and the sample preparation procedure.

First, we amended the column type and the detector to enhance GC–MS analysis conditions. Kawai et al^8^ employed a flame ionization detector and a nonpolar column (DB-1). In our validation experiments following their method, the BeOH peak exhibited tailing on the chromatogram when analyzed under their GC conditions. Consequently, we evaluated 3 different polar columns (Rxi17Sil-ms, VF-200 ms, and InertCap WAX-HT), all with the same column size (30 m × 0.25 mm, 0.25 μm) in order to select an appropriate column for BeOH determination. Among these, InertCap WAX-HT provided the best result, yielding a sharp and symmetrical peak of BeOH. However, InertCap WAX-HT required an extended analysis time to elevate the oven temperature to its maximum operational temperature; this was to prevent carryover by ensuring as many urinary endogenous substances as possible were eluted from the column. To address this issue, we used a fast GC column (DB-Heavy WAX) with the same polarity as InertCap WAX-HT. The analysis duration with DB-Heavy WAX was reduced to approximately 56% (17 minutes) of that required by Kawai et al’s^8^ method (30.25 minutes). Furthermore, we incorporated MS to enhance the detection sensitivity, selectivity, and identification ability.

**Figure 2:**
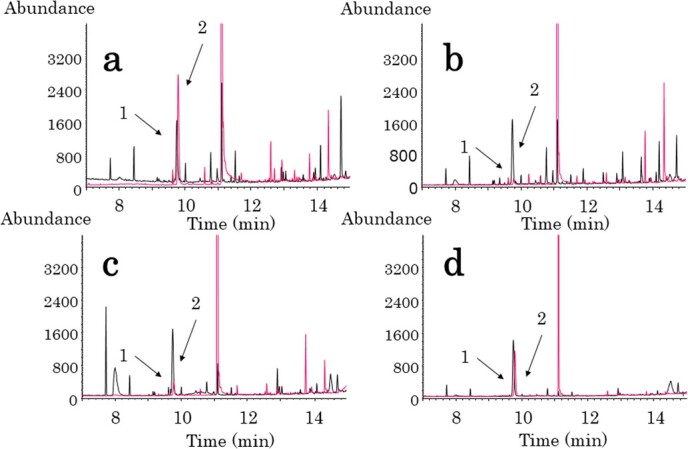
SIM chromatograms. Typical GC–MS chromatograms of urine (A) after adding BeOH to human urine (1000 μg/L), (B) control (48 μg/L), (C) of BeOH-exposed worker preshift (120 μg/L), and (D) of BeOH-exposed worker postshift (2553 μg/L: diluted 5 times). Peaks of (1:black:*m*/*z* = 85) benzyl-d_7_ alcohol (I.S.:500 μg/L) and (2:pink:*m*/*z* = 108) benzyl alcohol. BeOH, benzyl alcohol; GC–MS, gas chromatography–mass spectrometry, *m*/*z*, mass-to-charge ratio.

For further enhancement of the sample preparation procedure, we modified the I.S. substance, altered the extraction solvent, and introduced a washing step for the sample solution postextraction. Although Kawai et al^8^ used *cis*-3-methylcyclohexanol as an I.S. substance, an isotope dilution method was adopted using BeOH-d_7_ because the MS was used as the detector. Given the similar physicochemical properties of BeOH-d_7_ to BeOH, it (BeOH-d_7_) exerted an excellent correction effect for the matrix effect and recovery rate. Furthermore, the extraction solvent was converted from DIPE used by Kawai et al^8^ to MTBE to obtain a higher extraction rate. Consequently, the recovery rates (99%-106%) obtained with these improvements were higher than those of Kawai et al^8^ (90.3%). However, after continuously analyzing several samples, the BeOH peak on the chromatogram was observed to diminish gradually. We hypothesized that the HCl added for hydrolysis entered the sample solution postextraction and damaged the column during GC analysis. To this end, we introduced a washing step with 100 mM phosphate buffer (pH 7.0) to eliminate HCl from the sample solution postextraction. This additional step facilitated continuous analysis of numerous samples without column degradation.

### 3.2. Validation of developed GC–MS method for analysis of urinary BeOH

Calibration curves exhibited linearity in the concentration range of 5-1000 μg/L, with coefficients of determination exceeding 0.999. The LOD and LOQ were determined to be 1.3 and 5.0 μg/L, respectively. Recovery rates ranged from 99% to 106%. Accuracy and precision for the intraday assay were found to be within 95.0%-100.2% and 0.4%-9.7%, respectively, and for the interday assay, they were within 93.3%-99.9% and 0.6%-9.4%, respectively (Table 1). These values satisfied the established criteria of MHLW and FDA.^10^^,^^11^ Overall, the intraday and interday variabilities were low, indicating adequate reproducibility of the method. Typical GC–MS chromatograms are presented in Figure 2, displaying a sharp and symmetrical peak of BeOH without interference from endogenous compounds.

### 3.3. Personal exposure concentrations of BeOH

Results for the personal exposure monitoring are presented in Table 2. All but 3 subjects were exposed to BeOH concentrations higher than the MAK proposed by DFG.

### 3.4. Urinary concentrations of BeOH and HA in BeOH-exposed and nonexposed workers

**Figure 3:**
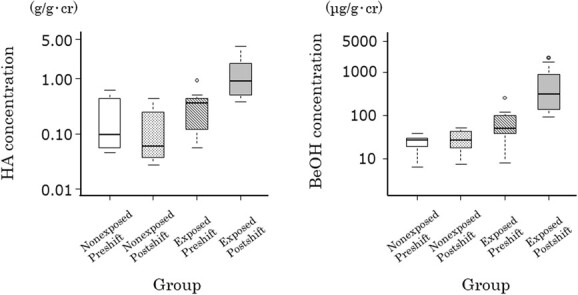
Boxplot of creatinine-corrected urinary concentrations of HA and BeOH in BeOH-exposed and -nonexposed workers. BeOH, benzyl alcohol; HA, hippuric acid.

Biological monitoring results are summarized in Table 2. Figure 3 illustrates a box plot of the Cr-corrected findings. BeOH and HA were detected in all urine samples collected from exposed workers. In the urine samples collected from nonexposed workers, BeOH was identified in 90% of preshift samples and in 100% of postshift samples, whereas HA was detected in 100% of preshift samples and 80% of postshift samples. In all urine samples, the HA concentration was significantly greater than that of BeOH. The GMs of BeOH and HA concentrations in postshift urine samples from exposed workers were significantly elevated compared with their preshift samples (BeOH, post-/pre-shift GM ratio = 7.5-7.8, *P* < .001; HA, post-/pre-shift GM ratio = 4.3-4.5, *P* < .001) and markedly higher than those in postshift samples of nonexposed workers (BeOH, exposed-/nonexposed-workers’ GM ratio = 14.8-19.0, *P* < .001; HA, exposed-/nonexposed-workers’ GM ratio = 12.1-15.3, *P* < .001), irrespective of correction for urine density. Conversely, the GMs of BeOH and HA concentrations in preshift urine samples from nonexposed workers did not differ significantly from their postshift samples (BeOH, *P* = .572 to .923; HA, *P* = .444 to .486), but they differed significantly from those in preshift samples of exposed workers, except for the observed and SG-corrected GMs of HA (BeOH, *P* = .034 to .045; HA, *P* = .069 to .137).

### 3.5. Correlation between personal exposure concentrations of BeOH and BeOH or HA concentrations in urine samples

**Figure 4:**
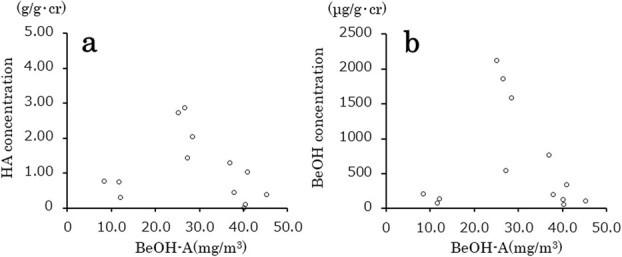
Scatter diagrams for the personal exposure concentrations of benzyl alcohol (BeOH-A) versus the differences between pre- and post-shift urine creatinine-corrected concentrations of (A) hippuric acid (HA-U) and (B) benzyl alcohol (BeOH-U), *n* = 13.

A simple regression analysis revealed that both biomarkers in the postshift urine samples had no significant correlation with the personal exposure concentrations of BeOH (BeOH-U [μg/L] = −6.11 × BeOH-A [mg/m^3^] + 798, *r* = −0.123, *P* = .688; HA-U [g/L] = 0.006 × BeOH-A [mg/m^3^] + 1.41, *r* = 0.056, *P* = .855), even after correction for Cr (BeOH-U [μg/g Cr] = −6.21 × BeOH-A [mg/m^3^] + 883, *r* = −0.098, *P* = .751; HA-U [g/g Cr] = −0.006 × BeOH-A [mg/m^3^] + 1.61, *r* = −0.069, *P* = .823) or SG (BeOH-U [μg/L)] = −5.96 × BeOH-A [mg/m^3^] + 779, *r* = −0.123, *P* = .688; HA-U [g/L] = 0.005 × BeOH-A [mg/m^3^] + 1.38, *r* = 0.054, *P* = .860). Moreover, the analysis based on the difference between pre- and post-shift urine concentrations instead of postshift urine concentrations, also found no correlation (without correction, BeOH-U [μg/L] = −4.89 × BeOH-A [mg/m^3^] + 677, *r* = −0.102, *P* = .741; HA-U [g/L] = −0.004 × BeOH-A [mg/m^3^] + 1.32, *r* = −0.044, *P* = .886; after correction for Cr, BeOH-U [μg/g Cr] = −5.12 × BeOH-A [mg/m^3^] + 778, *r* = −0.086, *P* = .780, HA-U [g/g Cr] = −0.014 × BeOH-A [mg/m^3^] + 1.50, *r* = −0.180, *P* = 0.556; after corretion for SG, BeOH-U [μg/L] = −4.75 × BeOH-A [mg/m^3^] + 660, *r* = −0.101, *P* = 0.742, HA-U [g/L] = −0.004 × BeOH-A [mg/m^3^] + 1.29, *r* = −0.048, *P* = .877]. Scatter diagrams for the personal exposure concentrations of BeOH versus the differences between pre- and post-shift urine concentrations of BeOH or HA are depicted in Figure 4.

**Table 2:** Personal exposure concentrations of benzyl alcohol (BeOH-A) and urinary benzyl alcohol (BeOH-U) and urinary hippuric acid (HA-U) concentrations in BeOH-exposed and -nonexposed workers.^a^

**Analyte**	**Correction for**	**Unit**	**Preshift**								**Postshift**								**Postshift vs preshift** ^**b**^			**Exposed workers vs nonexposed workers** ^**c**^					
																						**Preshift**			**Postshift**		
			**AM**	**ASD**	**GM**	**GSD**	**Range**			**Quantifiable, %**	**AM**	**ASD**	**GM**	**GSD**	**Range**			**Quantifiable, %**	**Ratio of GM**	**Ratio of GSD**	***P* value**	**Ratio of GM**	**Ratio of GSD**	***P* value**	**Ratio of GM**	**Ratio of GSD**	***P* value**
**Exposed workers (13 men)**																											
BeOH-A	—	mg/m^3^	—	—	—	—	—			—	29.3	12.4	26.1	1.7	8.4	—	45.2	100	—	—	—	—	—	—	—	—	—
BeOH-U	None (ie, as observed)	μg/L	85.4	64.0	57.7	2.9	7.1	—	221.8	100	618.9	613.0	430.5	2.4	120.3	—	2353	100	7.5	0.8	<.001	2.8	1.0	0.040	19.0	0.8	<.001
	Cr	μg/g Cr	73.4	66.0	49.1	2.8	8.2	—	257.6	—	701.1	785.0	382.7	3.2	93.7	—	2206	—	7.8	1.1	<.001	2.2	1.6	0.034	14.8	1.8	<.001
	SG (1.020)	μg/L	83.5	63.0	56.4	2.9	6.9	—	215.9	—	603.9	598.0	420.8	2.4	117.7	—	2303	—	7.5	0.8	<.001	2.8	1.0	0.045	19.0	0.8	<.001
HA-U	None	g/L	0.36	0.25	0.28	2.14	0.08	—	0.75	100	1.57	1.22	1.22	2.08	0.44	—	3.82	100	4.3	1.0	<.001	2.4	0.6	0.088	15.3	0.7	<.001
	Cr	g/g Cr	0.33	0.25	0.24	2.48	0.06	—	0.93	—	1.43	1.10	1.09	2.18	0.38	—	3.81	—	4.5	0.9	<.001	1.9	0.9	0.137	12.1	0.8	<.001
	SG (1.020)	g/L	0.36	0.25	0.28	2.14	0.08	—	0.73	—	1.53	1.18	1.19	2.07	0.43	—	3.71	—	4.3	1.0	<.001	2.4	0.6	0.069	14.9	0.7	<.001
**Nonexposed workers (10 men)**																											
BeOH-U	None (ie, as observed)	μg/L	30.8	24.0	20.6	3.0	<5.0	—	74.5	90	34.1	29.0	22.7	2.9	<5.0	—	94.9	100	1.1	1.0	.923	—	—	—	—	—	—
	Cr	μg/g Cr	25.3	10.0	22.5	1.8	5.4	—	39.7	—	29.5	14.0	25.8	1.8	7.5	—	51.9	—	1.1	1.0	.923	—	—	—	—	—	—
	SG (1.020)	μg/L	30.0	23.0	20.2	3.0	<5.0	—	72.5	—	33.2	28.0	22.2	2.9	<5.0	—	92.3	—	1.1	1.0	.572	—	—	—	—	—	—
HA-U	None	g/L	0.21	0.23	0.12	3.40	0.02	—	0.71	100	0.14	0.15	0.08	3.07	<0.020	—	0.48	80	0.7	0.9	.444	—	—	—	—	—	—
	Cr	g/g Cr	0.21	0.22	0.13	2.72	0.05	—	0.61	—	0.15	0.15	0.09	2.87	0.03	—	0.44	—	0.7	1.1	.486	—	—	—	—	—	—
	SG (1.020)	g/L	0.21	0.22	0.12	3.38	0.02	—	0.69	—	0.13	0.15	0.08	3.06	<0.020	—	0.47	—	0.7	0.9	.462	—	—	—	—	—	—

Abbreviations: AM, arithmetic mean; ASD, arithmetic standard deviation (dimensionless); Cr, creatinine concentration; GM, geometric mean; GSD, geometric standard deviation (dimensionless); LOQ, limit of quantification (5 μg/L for BeOH and 0.02 g/L for HA, respectively); *n* < LOQ, number of values below LOQ; SG, urine specific gravity.

a The data below the LOQ for BeOH and HA were set to half the LOQ in the calculations of GM and GSD.

b *t* test for paired samples.

c Welch *t* test.

## 4. Discussion

In this study, we discuss the biological monitoring results of workers involved in removing paint films from bridges using a paint remover containing BeOH. We devised a GC–MS methodology for analyzing urinary BeOH and measured BeOH and HA in the urine of exposed and nonexposed workers. We evaluated cross-shift changes within each participant group and differences between the groups, and correlation with personal exposure for these biomarkers. To the best of our knowledge, this is the first study focusing on biomarkers of occupational exposure to BeOH.

The obtained results indicated 6 key findings: (1) BeOH and HA were quantified in nearly all urine samples; (2) in all urine samples, the HA concentration was much higher than the BeOH concentration; (3) the post-/pre-shift GM ratios of BeOH and HA in urine samples acquired from exposed workers were >7 and >4, respectively; (4) the exposed-/nonexposed-workers’ GM ratios of BeOH and HA in postshift urine samples were >14 and >12, respectively; (5) the post-/pre-shift GM ratio and exposed-/nonexposed-workers’ GM ratio (in postshift urine samples) of BeOH were higher than those of HA; (6) no correlation was observed between personal exposure concentrations of BeOH and either BeOH or HA concentrations in urine samples.

Significant increases were observed in urinary BeOH and HA concentrations between pre- and post-shift, suggesting that none of the workers was effectively protected from exposure to BeOH, which would indicate that either respiratory tract absorption, dermal absorption, or both occurred. Dermal absorption is particularly important for BeOH, which has been assigned an “H” by the DFG^2^ because skin contact with BeOH potentially significantly contributes to its systemic toxicity for an organism. The biological monitoring of substances that can be absorbed through the skin—such as BeOH—is useful in assessing whether a worker has been exposed to the substance because it reflects the amount of the substance taken into the body, regardless of the route of exposure and whether the worker was wearing protective equipment. If it is clear that BeOH has been taken into the body of a worker, even if BeOH poisoning did not occur, wearing appropriate protective equipment is essential to prevent BeOH poisoning.

Conversely, no correlation was observed between personal exposure concentrations of BeOH and either BeOH or HA concentrations in urine samples. This may be because the factors influencing urine concentrations include not just personal exposure concentrations but also differences in the effectiveness of protective equipment for each worker. Therefore, the biomarker concentrations that correspond to the MAK (22 mg/m^3^) proposed by the DFG could not be estimated. In addition, the biomarkers that correspond to the OEL-C (25 mg/m^3^) proposed by the JSOH could not be estimated because no measurements were taken to determine ceiling levels. Although further studies are needed to establish the occupational exposure limit based on biological monitoring that also accounts for dermal exposure, our results at least suggest that both urinary BeOH and HA could potentially serve as biomarkers of occupational exposure to BeOH.

Intriguingly, although the concentration of BeOH was markedly lower than that of HA in each urine sample, the post-/pre-shift GM ratio and exposed-/nonexposed-workers’ GM ratio of BeOH (in postshift urine samples) surpassed those of HA. One possible explanation for this observation could be the different magnitudes of increase in BeOH and HA due to dietary intake. Previous studies^22^^-^^24^ have highlighted that specific foods significantly augment urinary HA concentrations. For instance, chlorogenic acid in coffee, quinic acid in cranberries, kiwifruit, and prunes, and benzoic acid in soft drinks are metabolized in the body to produce HA (Figure 1). Alternatively, BeOH is a common component in various foods such as liqueurs, aromatized wines, wine-based drinks, wine-product cocktails, chocolate, and fine bakery wares as well as in natural components like wines, grapes, sour cherries, cider, mushrooms, chestnuts, almonds, and cloves.^25^ The dietary exposure to BeOH from its use as a food additive has been calculated based on maximum permitted levels recommended in the European Union legislation, estimated to range from 0.05 to 0.37 mg/kg/d in adults (18-64 years).^25^ As most of BeOH ingested from those foods is metabolized to HA via benzoic acid^7^ (Figure 1), the concentration of BeOH detected in urine is considered to be extremely low. The variations in the effects of BeOH and HA on dietary intake were supported by the present results obtained from nonexposed workers (GM of BeOH, 20.6-22.7 μg/L; GM of HA, 0.08-0.12 g/L). Furthermore, the present findings were similar to those of Kawai et al^8^ and Inoue et al^26^ for toluene-nonexposed workers (GM of BeOH reported by Kawai et al = 53 μg/L; GMs of BeOH and HA reported by Inoue et al = 55 μg/L and 0.224 g/L, respectively). Kawai et al^8^ reported that, in contrast to HA, the ingestion of benzoate-containing soft drinks did not increase urinary BeOH concentrations in volunteers. These insights suggest that the impact of dietary intake on elevated urinary concentrations is more pronounced for HA than for BeOH, which may have partially masked the contribution of BeOH exposure. The differences in BeOH concentrations in preshift urine samples of exposed and nonexposed workers can be potentially attributed to dietary intake. However, the specific implications of this observation could not be conclusively established in this study.

Another possible contributing factor could be the influence of ALDH genetic polymorphism on the metabolism of BeOH. Previous research by Kawamoto et al^27^^-^^29^ reported the effects of ALDH2 genetic polymorphism (low-*K*_m_ aldehyde dehydrogenase) on toluene metabolism. They discovered that workers with a homozygous genotype of an inactive ALDH2 enzyme metabolized less HA from toluene than workers with a homozygous genotype of a normal ALDH2 or with a heterozygous genotype of both normal and inactive ALDH2. This conclusion was drawn from comparing differences in regression line slopes between personal exposure to toluene and urinary HA levels among these groups. The effects of ALDH2 genetic polymorphism on toluene metabolism are believed to influence BeOH metabolism similarly (Figure 1). Therefore, although the proportion of each ALDH2 genotype among exposed workers was not accessible in this study, this proportion is suspected to be a key factor in characterizing the GM of HA in postshift urine samples of these workers. In conclusion, BeOH appears to be a superior biomarker of occupational exposure to BeOH than HA because it is less influenced by dietary intake and the genetic polymorphism of ALDH2.

Nevertheless, this study has certain limitations. We were unable to evaluate the effects of various confounding factors and their interactions, including personal and dermal exposure levels to BeOH, ALDH2 genotype, dietary habits, age, smoking habits, and alcohol consumption habits. Dermal exposure levels in particular warrant attention, given that BeOH can be absorbed through the skin.^2^ The presence of these confounding factors may have produced inaccurate results.

Therefore, further research is required to accurately assess the utility of BeOH and HA as biomarkers of occupational exposure to BeOH.

## 5. Conclusions

This research developed a reliable and improved GC–MS method for the detection of urinary BeOH and effectively used it in the analysis of urine samples collected from exposed and nonexposed workers. The biological monitoring results suggest that urinary BeOH and HA hold potential as biomarkers for occupational exposure to BeOH. More specifically, BeOH may be a superior biomarker to HA because it demonstrates less sensitivity to confounding factors such as dietary intake and the genetic polymorphism of ALDH2. This study, although informative and pioneering, does not incorporate several potential confounding factors, such as personal and dermal exposure levels of BeOH and genetic variation in ALDH2. This necessitates further comprehensive research to investigate these influences for assessing the role of BeOH and HA as occupational biomarkers.

Despite these limitations, this research considerably contributes to the understanding of biomarkers for BeOH exposure. It holds substantial relevance for industry safety measures and health care, offering practical tools to mitigate occupational exposure and potentially transform safety protocols in relevant workplaces.

## Data Availability

The data cannot be shared due to privacy and ethical reasons.
